# Bioactive peptides in the pancreatin-hydrolysates of whey protein support cell proliferation and scavenge reactive oxygen species

**DOI:** 10.1080/19768354.2022.2130425

**Published:** 2022-10-11

**Authors:** Haesoo Jung, Damin Jung, Jaehoon Lee, Woojin Ki, Jung-Min Lee, Eun-Mi Kim, Myoung Soo Nam, Kee K. Kim

**Affiliations:** a Department of Biochemistry, College of Natural Sciences, Chungnam National University, Daejeon, Republic of Korea; b Division of Animal Resource Science, Chungnam National University, Daejeon, Republic of Korea; c Department of Predictive Toxicology, Korea Institute of Toxicology, Daejeon, Republic of Korea

**Keywords:** milk, whey protein, hydrolysis, inflammation, cellular senescence

## Abstract

Whey protein (WP) in milk shows physiologically active functions such as cholesterol control and immune system strengthening. In this study, we performed hydrolysis and peptide polarity fractionation to enhance the efficacy and diversity of its physiological activities, using the digesting enzyme, pancreatin. Our results indicate that hydrolysis significantly increased the cell proliferation of the WP fractions, with the lower-polarity fractions showing greater efficacy in this regard. Our results indicate that hydrolysis significantly increases cell proliferation of the WP fractions. Additionally, we confirmed differences in the antioxidant activity of the WP fractions as a function of polarity was confirmed via scavenging 2,2’-azino-bis (3-ethylbenzothiazoline-6-sulfonic acid) (ABTS) assay *in vitro*. WP itself did not show anti-inflammatory efficacy. However, all the hydrolyzed fractions downregulated the mRNA expression levels of inflammatory cytokines in all treated cell lines and, based on a senescence-associated (SA)-β-galactosidase assay, the fraction with the lowest polarity (F6) inhibited cellular senescence to the greatest extent. Furthermore, we identified the peptide sequences with various physiological activities from whey protein hydrolysates through mass spectrometry. Taken together, our results indicate that the fractionation of WP via hydrolysis generates novel functions including promoting cellular cell proliferation, anti-inflammatory effects, and enhancing antioxidant and anti-cellular senescence.

## Introduction

Milk is considered a nutritionally important food given that it contains essential nutrients for the body, such as proteins, calcium, and minerals (Paul et al. 2020). It is also well known that the continuous intake of calcium and the essential amino acids in milk prevent obesity (Abreu et al. 2012; Satija et al. 2013), type 2 diabetes, and hypertension (Martini and Wood 2009; McGrane et al. 2011). Moreover, the proteins in milk (80% casein protein and 20% whey protein (WP)) (Jeong et al. 2021) are known to be effective in enhancing gastrointestinal activity, function, and immunity (Stelwagen et al. 2009; Maldonado Galdeano et al. 2011). Furthermore, the amino acids absorbed following the breakdown of casein protein via digestion increase muscle mass and suppress hunger (Scognamiglio et al. 2004), and WP, which predominantly consists of α-lactalbumin (α-LA), β-lactoglobulin (β-LG), immunoglobulin (Ig), and bovine serum albumin (BSA) (Smithers et al. 1996), is digested and converted into amino acids faster after ingestion than casein protein (Koopman et al. 2009).

WP is rich in the essential amino acids cysteine and methionine as well as in branched-chain amino acids and tryptophan (Hulmi et al. 2010). Thus, it exhibits various physiologically active functions, such as cholesterol control and strengthening immune system strengthening (Badr et al. 2012; Niitsu et al. 2016; Bell et al. 2020). Specifically, cysteine, which is one of the constituent amino acids of glutathione, is involved in the body's antioxidant and immune systems (Iyer et al. 2009), and reportedly plays a critical role in the elderly who have reduced muscle mass and muscle strength due to insufficient protein intake (Bell et al. 2020). Currently, several studies are being conducted to identify low-molecular-weight peptides from milk proteins using hydrolytic enzymes to enhance the physiological activities of milk proteins and search for novel active peptides (Gomes-Santos et al. 2015; Kelly et al. 2016; Tasaka et al. 2018; Hinnenkamp et al. 2021). As a representative example, a bioactive peptide derived from buffalo casein, VLPVPQK, shows anti-aging and anti-osteopenic effects (Mada et al. 2017).

Cells, the basic functional units of all living organisms, are exposed to various internal and external stressors, when subjected to environmental changes, they regulate various physiological responses to maintain homeostasis, which if disrupted, results in the occurrence of many diseases (Ishikawa et al. 2008). For example, during the oxidative phosphorylation process for ATP production in mitochondria, free radicals and reactive oxygen species (ROS), which induce cellular oxidative stress, are generated (Sosa et al. 2013; Zorov et al. 2014; Rodney et al. 2016). These ROS then induce the denaturation of biomolecules via irreversible chemical reactions with various biomolecules, resulting in the occurrence of various diseases, such as those associated with inflammatory responses and accelerated aging (Cai and Harrison 2000; Nunomura et al. 2001; Trachootham et al. 2009). Therefore, antioxidants externally supplied through food may be necessary to eliminate excessive free radicals and ROS production.

In this study, we analyzed the diversity and efficacy of the physiological activity of WP fractions hydrolyzed using the digesting enzyme pancreatin. Specifically, the hydrolyzed WP fractions were separated based on peptide polarity and their bioactivities to different cell lines were investigated.

## Materials and methods

### Preparation of hydrolyzed WP and peptides

For WP hydrolysis, pancreatin (Bision Co. Ltd., Gyunggi-do, Korea) was added to WP at a ratio of 1:100. Thereafter, they were allowed to react for 5 h at 50°C in a shaking incubator (130 rpm) after which the hydrolysate was centrifuged at 12,000 g for 20 min at 40°C. This WP hydrolysate was then analyzed using a reversed-phase HPLC system (Waters Associates, Milford, MA., USA) with a C18 column (4.6 × 250 mm, Vydac, Hesperia, CA., USA) equilibrated with solvent A (0.1% trifluoroacetic acid [TFA] in deionized water) and eluted with a linear gradient of solvent B (0.1% TFA in acetonitrile) for 40 min. The experiment was performed at 25°C with the HPLC system at a flow rate of 1 ml/min, and the absorbance of the column elute was measured at 214 nm. Additionally, the hydrolyzed WP fractions (F1–F6) were also analyzed using a Multi Preparative HPLC system (YMC, Kyoto, Japan) with a C18 column (250 × 20.0 mm, YMC) equilibrated with solvent A and eluted with a linear gradient of solvent B for 60 min. The experiment was performed at 25°C using at a flow rate of 15 mL/min, and the absorbance of the column elute was measured at 214 nm. The hydrolyzed WP fractions were obtained based on the acetonitrile concentration (1–31%). The components of F6 were analyzed using liquid chromatography-mass spectrometry (LC-MS) in an EASY-nLC 1000 system (Thermo Fisher Scientific Inc., MA, USA). The peptide sequence present in the protein sequence data (NCBI) was confirmed using MASCOT (Matrix Science, London, U.K.; version 2.2.04). Peptides with significance were considered and analyzed using the MASCOT score value. Peptides identified in F6 of hydrolyzed WP were synthesized by Peptron, Inc. (Daejeon, South Korea).

### Cell culture and reagent

Human lung adenocarcinoma cell line A549, human liver hepatocellular carcinoma cell line HepG2, murine macrophage cell line RAW 264.7, and primary human dermal fibroblast (HDF) cell line (Cell Applications, San Diego, CA, USA) were maintained in Dulbecco’s modified Eagle’s medium (DMEM; Welgene, Gyeongsangbuk-do, Korea) supplemented with 10% heat-inactivated fetal bovine serum (FBS; Gibco, Waltham, MA., USA) and 1% penicillin/streptomycin (Corning Inc., Corning, NY., USA) at 37°C in a humid 5% CO_2_ atmosphere. Resveratrol was purchased from Sigma-Aldrich (St Louis, MO., USA), and the senescence-associated-β-galactosidase (SA-β-gal) staining kit was purchased from Cell Signaling Technology (Beverly, MA., USA).

### ABTS radical scavenging activity

The antioxidant activities of the WP fractions were measured using the 2,2'-azino-bis-3-ethylbenzothiazoline-6-sulphonic acid (ABTS) assay (Cambronero-Urena et al. 2021). Specifically, the ABTS + radical solution was prepared by reacting a 7 mM ABTS solution with 2.4 mM potassium persulfate solution at 25°C for 24 h. The hydrolyzed WP was then reacted with the prepared ABTS + radical solution for 4 min, after which the absorbance was measured at 650 nm using a microplate reader (Molecular Devices. San Jose, CA., USA). Antioxidant activity was calculated using the following equation:

$$ABTS\;radical\;scavenging\;activity\;(\text{\%})=\left[\frac{\;A_{control\;}-\;A_{sample}\;}{\;A_{control}}\right]\times 100$$

Where A is the absorbance of ABTS radical in distilled water; Ais the absorbance of an ABTS radical solution mixed with sample.

### Cell proliferation measurement

Cell proliferation was determined via MTS assay. A549, HepG2, and RAW 264.7 cells were seeded at 3,000 cells per well in 96-well plates and incubated for 24 h. Thereafter, the cells were treated with hydrolyzed WP fractions at concentrations of 0.05, 0.25, and 1.25 mg/mL followed by incubation for 36 h. Cell proliferation was then analyzed using a Cell Titer 96R Aqueous One Solution Cell Proliferation Assay (Promega, Madison, WA., USA), and absorbance was measured at 490 nm using a microplate reader (Molecular Devices). Finally, mitochondrial activity cell proliferation was calculated using the following equation:

$$Cell\;proliferation\;(\text{\%})=\left[\frac{A_{sample}}{A_{control}}\right]\times 100$$

### RNA isolation and quantitative RT–PCR (qRT-PCR) analysis

RAW 264.7 cells were seeded at 1 × 10^5^ cells per well in 6-well plates and incubated for 24 h. Thereafter, the cells were treated with the hydrolyzed WP fractions and incubated for 30 h followed by lipopolysaccharide (LPS) treatment at a concentration of 1 μg/mL for 6 h. Total RNA was then isolated from the RAW 264.7 cells using the GeneAll Hybrid-R RNA Purification Kit (GeneAll, Seoul, Korea). Thereafter, cDNA was synthesized using a random hexamer and M-MLV reverse transcriptase (Promega), and qRT-PCR was performed using the AriaMx real-time PCR system (Agilent Technologies, Santa Clara, CA., USA) (Jung et al. 2022). The primers used in this study are listed in Supplementary table 1.

### Senescence-associated-β-galactosidase (SA-β-gal) activity analysis

HDFs were seeded at 2 × 10^5^ cells per well in 6-well plates and incubated for 24 h. This was followed by pretreatment with 1.25 mg/mL of the hydrolyzed WP fractions and incubation for 24 h. Thereafter, senescence was induced via treatment with 600 μM H_2_O_2_ for 2 h, and the cells were allowed to recover for a period of 4 days. After washing twice with PBS, the cells were fixed with 1× fixative solution, and SA-β-gal staining was performed using a senescence-β-galactosidase staining kit (Cell Signaling Technology). Finally, the H_2_O_2_-treated HDFs were photographed using a light microscope (Nikon, Tokyo, Japan), and the number of X-gal-stained cells was counted against the total number of HDFs and expressed as a percentage of SA-β-gal-positive cells.

### Statistical analysis

Data were expressed as mean ± standard deviation (SD). All experiments were independently performed in triplicate, and statistical significance was determined using the Student’s t-test. *p < 0.05 was considered statistically significant.

## Results

### HPLC chromatogram of hydrolyzed WP

**Figure 1.**
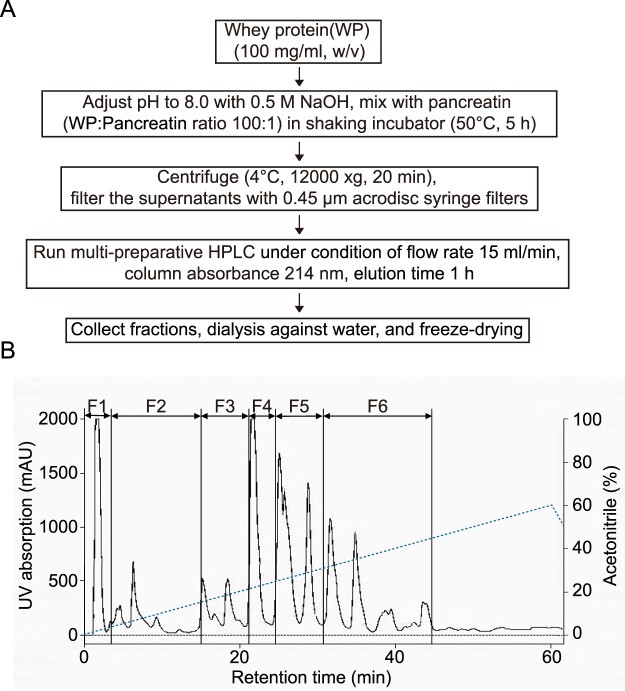
HPLC chromatogram of hydrolyzed whey protein (WP). (A) WP hydrolyzed using pancreatin. Chromatogram of (B) WP and (C) hydrolyzed WP. Fractions were obtained according to acetonitrile concentration (1–31%). Elution time, 40 min; Flow rate, 1 mL/min; UV detection wavelength, 214 nm.

Pancreatin is a mixture of carbohydrates, lipids, and protein-digesting enzymes. Specifically, it contains various proteases, including trypsin, chymotrypsin, elastase, and carboxypeptidase A and B (Rothenbuhler and Kinsella 1986). The complex action of these pancreatin enzymes produce various types of degradation products from their substrates. Via enzymatic hydrolysis and fractionation as determined using a multi-preparative HPLC system (repeated six times), we determined the functionality of the hydrolysate produced by the action of pancreatin on WP (Figure 1(A)), and separated the hydrolyzed WP-driven peptides based on their polarity via multi-preparative HPLC led to the observation of several peaks (Figure 1(B,C)). Six reproducible peaks were selected from repeated trials, and fractions were obtained at each detected acetonitrile concentration (F1, 1%; F2, 3%; F3, 15%; F4, 20%; F5, 25%; and F6, 31%). Further, each of the six fractions obtained was dialyzed against water to remove acetonitrile, and thereafter, freeze-dried, and stored at −20°C until later use.

### Enhancement of the cellular cell proliferation of WP via hydrolysis

**Figure 2.**
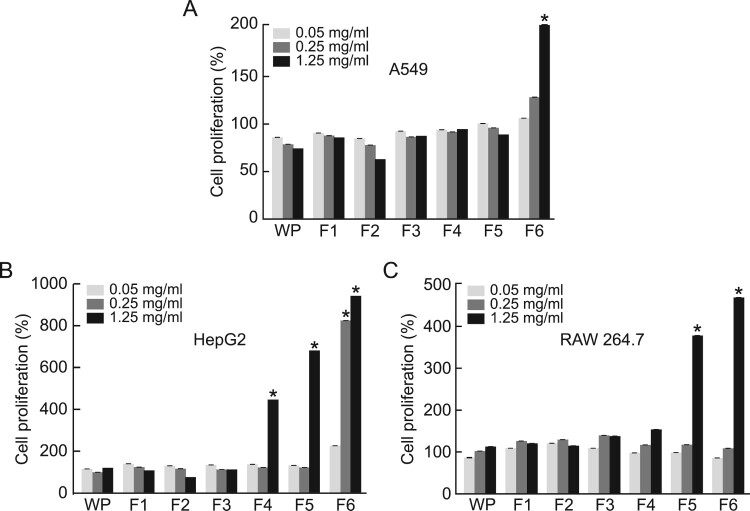
Effect of hydrolyzed WP on cell proliferation. (A) A549, (B) HepG2, and (C) RAW 264.7 cells treated with the indicated concentrations of hydrolyzed WP for 36 h. Cell proliferation was measured via MTS assays. The results are presented as mean ± S.E.M relative cell proliferation compared with the control group. *p < 0.05, compared with non-treated control.

To examine whether hydrolysis influenced the effect of WP on cell proliferation, the effects of the treatment of different cell lines with the hydrolyzed WP fractions at concentrations of 0.05, 0.25, and 1.25 mg/mL for 36 h were determined via MTS assay. No changes in the cell proliferation in the different cell lines were observed following the F1, F2, and F3 WP treatments. However, the F6 treatment induced an increase in cell proliferation in all the treated cell lines, whereas the F5 treatment enhanced cell proliferation only in the HepG2, RAW 264.7, and A549 cell lines (Figure 2(A-C). Further, the F4 treatment enhanced cell proliferation in HepG2 cells only. These results suggest that cell proliferation enhancing peptides can be generated via the hydrolysis of WP.

### Antioxidant activity of hydrolyzed WP

ROS are unstable high-energy, and highly reactive molecules that contain oxygen atoms and unpaired electrons. When their levels exceed a particular threshold in cells, they eventually inhibit cell homeostasis by attacking biomolecules such as DNA, RNA, proteins, and lipids, and ROS-induced decline in cellular function causes various diseases, including aging (Kikuchi et al. 2002; Kennedy et al. 2005; Nunomura et al. 2006).

**Figure 3.**
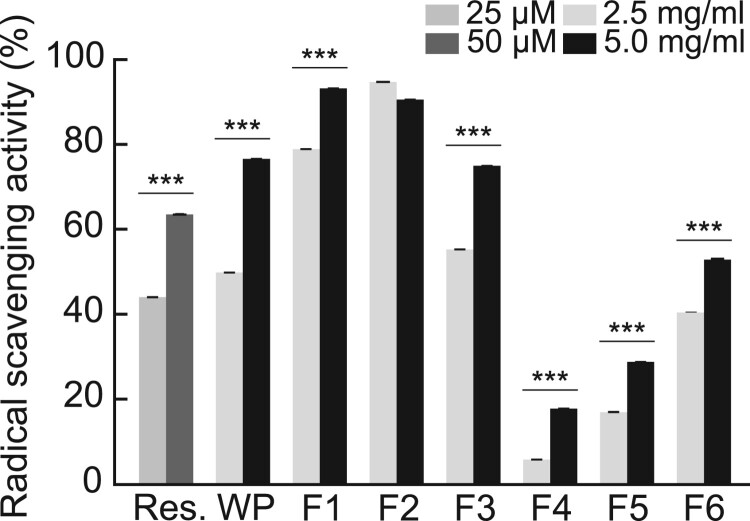
Antioxidant activity of hydrolyzed WP. Radical scavenging activity of hydrolyzed WP measured at the indicated concentrations using an ABTS + radical scavenging assay. Resveratrol (Res.) was used as the positive control. ***p < 0.001.

Therefore, we examined the antioxidant activity of the hydrolyzed WP fractions against free radical scavenging activity via ABTS assay. Resveratrol, which is known to show antioxidant activity, was used a positive control. We observed that the antioxidant activities of WP fractions at concentrations of 2.5 and 5.0 mg/mL were similar to those of resveratrol at 25 and 50 μM concentrations, respectively. Additionally, at a concentration of 5.0 mg/mL, treatments F1, F2, and F3 showed excellent radical scavenging activities (95.7 ± 1.60, 93 ± 0.98, and 76.9 ± 4.36%, respectively) (Figure 3(A)). Even though the treatment with F4, F5, or F6 showed lower antioxidant activities than the other fractions, our results indicate that their concentration-dependent increase in radical scavenging activity was comparable to those of the other hydrolyzed WP treatments.

### Anti-inflammatory activity of hydrolyzed WP

**Figure 4.**
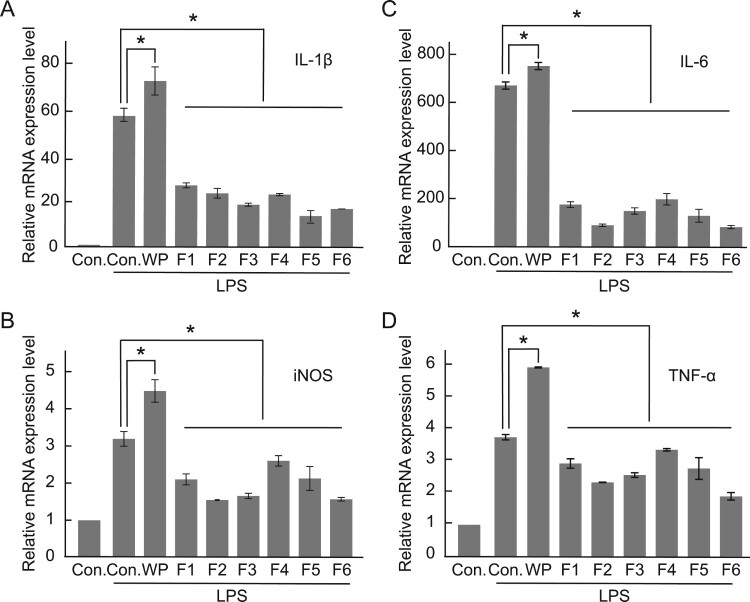
Anti-inflammatory activity of hydrolyzed WP. RAW 264.7 cells were treated with hydrolyzed WP for 36 h and thereafter, treated with LPS to induce an inflammatory response. The mRNA expression levels of (A) IL-1β, (B) IL-6, (C) iNOS, and (D) TNF-α measured via qRT-PCR. The results are presented as mean ± S.E.M. *p < 0.05, compared with the respective LPS-treated controls. Con., control.

Considering the role of ROS in inflammation in cells, the increase in the antioxidant activity of WP following hydrolysis inspired us to investigate its efficacy for inflammatory reactions. To this end, the expression levels of the mRNAs of representative inflammatory cytokines, TNF-α and IL-6, and a nitric oxide-generating enzyme, iNOS, were examined in RAW 264.7 cells (Dinarello 2000). We observed that WP treatment upregulated the LPS-induced increase in the expression levels of the mRNA of inflammatory cytokines; however, all the fractions obtained via hydrolysis effectively downregulated the expression of inflammatory cytokines (Figure 4(A-D)). In particular, F6, which induced the highest increase in cell proliferation in the different cell lines, effectively suppressed the expression of all inflammatory cytokines. Notably, all the hydrolyzed WP treatments downregulated the mRNA expression levels of all inflammatory cytokines. These results suggest that it is possible to obtain fractions containing anti-inflammatory peptides from WP via hydrolysis.

### Suppression of cellular senescence by hydrolyzed WP

**Figure 5.**
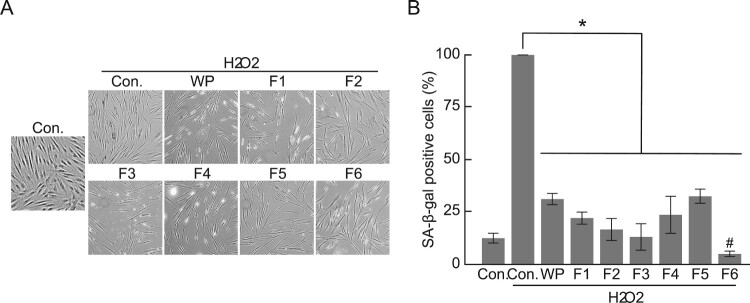
SA-β-galactosidase activity (SA-β-gal) of hydrolyzed WP. (A) HDFs treated with hydrolyzed WP for 24 h, followed by the induction of senescence using H_2_O_2_. The level of senescence in HDFs was measured using SA-β-gal assay. Senescent cells (SA-β-gal positive cells) were stained blue using X-gal. (B) Percentage of SA-β-gal positive cells. The results are presented as mean ± S.E.M. *p < 0.05, compared with the respective H_2_O_2_-treated control cells. # < 0.05, compared with the control cells (non-H_2_O_2_-treatment).

The antioxidant and cell proliferation enhancing effects of the hydrolyzed WP fractions prompted us to examine whether these hydrolyzed WP fractions affected cellular senescence. In this regard, we used the well-known biomarker, SA-β-gal, to detect senescent and aging cells and confirm the degree of cellular senescence. As expected, H_2_O_2_ treatment effectively induced the senescence of HDFs by increasing the number of β-gal-positive cells (Figure 5). Further, we observed that treatment with WP and F1–F5 reduced the proportion of β-gal-positive cells from approximately 25–12.5%. Interestingly, the decrease in the proportion of β-gal-positive cells by F6 treatment was confirmed to be 5%, i.e. less than 12.5% indicating that it suppressed senescence induced by H_2_O_2_. This infers that the F6 treatment showed more effective inhibitory activity than the basal level of cellular senescence even in the induction of cellular senescence by the H_2_O_2_ treatment. Therefore, hydrolysis can enhance the inhibitory activity of WP on cellular senescence.

### Physiological activity of peptides identified in F6

We performed LC-MS analysis to identify the peptides of F6 that showed significant antioxidant and anti-aging activities among the hydrolyzed WP. We confirmed that F6 contained two main peaks through the HPLC results. As a result of calculating the area of each peak as a percentage, the peak with high polarity was 40.62% and the second peak occupied 28.94%. Furthermore, of the 18 peptides detected through LC-MS analysis of F6, it was confirmed that VLVLDTDYKK (P1) originated from β-lactoglobulin accounted for 17.57% of all the peptides in F6 and VGINYWLAHK (P2) originated from α-lactalbumin accounted for 16.22% of all the peptides in F6 (Table 1). These two peptides are the most abundant ones in F6.

**Table 1.** Peptides identified in F6 of hydrolyzed WP.

No	Protein name	Sequence	Molecular weight (Da)	Peptide sequence%	References
1	β-Lactoglobulin	VLVLDTDYKK ALPMHIR + Oxidation (M) IDALNENKVLVLDTDYKK LIVTQTMK ALPMHIR	18555	17.57 5.41 2.70 2.70 2.70	Wang et al. (2021), Dyer et al. (2016), Wang et al. (2014), Gurbuz et al. (2015)
2	α-Lactalbumin	VGINYWLAHK FLDDDLTDDIMCVK IWCKDDQNPHSSNICNISCDK ILDKVGINYWLAHK ALCSEKLDQWLCEK	14633	16.22 12.16 4.05 4.05 2.70	Gurbuz et al. (2015), Wang et al. (2021), Boehmer et at. (2008) (2008)
3	Serum albumin	LSQKFPK KVPQVSTPTLVEVSR FKDLGEEHFK KQTALVELLK	68416	10.81 2.70 2.70 2.70	Li et at. (2007), Luna et al. (2014)
4	Lactoferrin	KGSNFQLDQLQGR LRPVAAEIYGTK	77129	2.70 2.70	Dyer et at. (2016) Dyer (2016)
5	Butyrophilin	VSLVEDHIAEGSVAVR VAALGSDPHISMK	59892	2.70 2.70	Mather. (2000)Smolenski et al. (2007)

**Figure 6.**
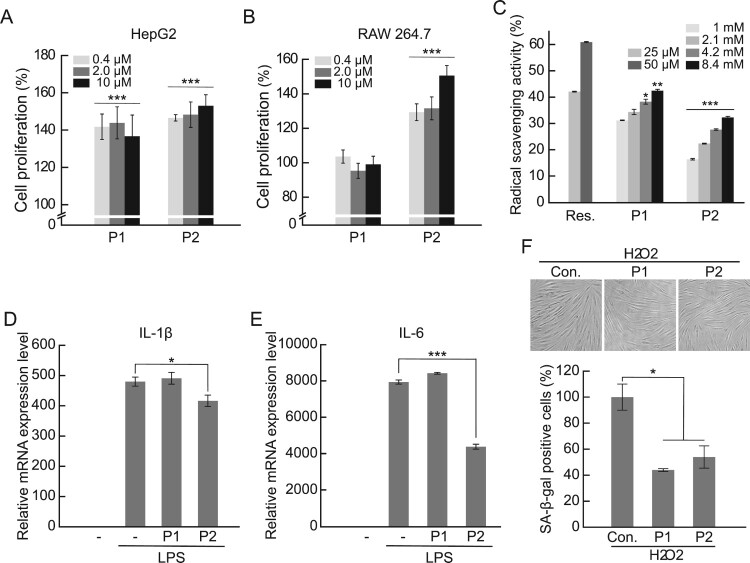
Physiological activities of WP hydrolysates-derived peptides P1 and P2. (A) HepG2 and (B) RAW 264.7 cells treated with the indicated concentrations of VLVLDTDYKK (P1) and VGINYWLAHK (P2) for 36 h. Cell proliferation was measured by MTS assays. ***p < 0.001, compared with the non-treated control. (C) Radical scavenging activity of P1 and P2 measured at the indicated concentrations using an ABTS + radical scavenging assay. Resveratrol was used as the positive control. (D,E) RAW 264.7 cells were treated with P1 and P2 for 36 h and thereafter, treated with LPS to induce an inflammatory response. The mRNA expression levels of (D) IL-1β, (E) IL-6 were measured by qRT-PCR. The results are presented as mean ± S.E.M. *p < 0.05, ***p < 0.001, compared with the respective LPS-treated controls. (F) HDFs treated with P1 and P2 for 24 h, followed by the induction of senescence using H_2_O_2_. The level of senescence in HDFs was measured using a SA-β-gal assay. Senescent cells (SA-β-gal positive cells) were stained blue using X-gal. (G) Percentage of SA-β-gal positive cells. The results are presented as mean ± S.E.M. *p < 0.05, compared with the respective H_2_O_2_-treated control cells.

Next, we investigated whether the various physiological activities of F6 were attributed to P1 and P2. In both HepG2 and RAW 264.7 cells, which showed a substantial increase in cell proliferation by F6 (Figure 2), P2 showed the highest activity, whereas P1 increased cell proliferation only in HepG2 cells (Figure 6(A,B)). The radical scavenging activities of P1 and P2 were similar to the concentration-dependent increase of F6. Furthermore, to confirm the contribution of P1 and P2 to the anti-inflammatory activity of F6, we examined the inhibitory activity of P1 and P2 on IL-1β and IL-6 and found that F6 had a marked inhibitory effect on inflammatory cytokines. we examined P1 and P2 for their effects on IL-1β and IL-6 gene expression and found that both P2 had a marked inhibitory effect on inflammatory cytokines.

As a result, P1 showed no inhibition of the expression of both inflammatory cytokines induced by LPS, whereas P2 showed significant inhibitory activity on the expression of IL-1β and IL-6 (Figure 6(D,E)). Finally, in order to determine the activity of P1 and P2 on cellular senescence, we investigated the inhibitory effect on cellular senescence induced by H_2_O_2_ treatment. Despite weaker effect than F6, approximately 50% of inhibitory activity against cellular senescence was confirmed in both P1 and P2 (Figure 6(F,G)). These results suggest that both P1 and P2 considerably contribute to the effects of hydrolyzed WP on various physiological activities, including anti-cellular senescence.

## Discussion

In this study, we obtained WP fractions via hydrolysis based on fractionation according to polarity and investigated the physiological activity of these fractions. WP is rich in essential amino acids, such as cysteine and methionine, and is known to have various advantages, such as immune regulation (Badr et al. 2012), weight loss (Pezeshki et al. 2015), and blood sugar control (Hussein et al. 2020). In our previous study, we confirmed that β-lactoglobulin, which accounts for 50–55% of WP, inhibits cellular senescence by inhibiting oxidative stress in cells (Kim et al. 2019). The hydrolysates of WP have the same amino acid composition as WP; however, they absorb plasma amino acids faster than WP, promote insulin secretion, lower blood sugar levels, and enhance muscle growth (Moro et al. 2019). Although several studies have reported the effect of WP on the physiological activity of cells, the number of studies focusing on the effects of WP hydrolysates is limited (Lollo et al. 2011). To maximize the positive effects of the physiological activity of WP hydrolysates, the enrichment of the bioactive components via fractionation is essential, as confirmed in this study.

Specifically, to maximize the efficacy of physiological activity, we hydrolyzed WP via the action of the enzyme mixture pancreatin, and obtained WP fractions as a function of the polarity of the constituent peptides. Our results indicated that fractions with low peptide polarity, especially F6 with the lowest polarity, significantly enhanced cell proliferation in all the cell lines investigated (Figure 2). These results suggest that hydrolysis induces a decrease in the polarity of the proteins constituting WP, and that peptide molecules with low polarity can promote the cell proliferation of cells. Additionally, in terms of the antioxidant activity of the WP-derived peptide fractions, we observed that fractions containing low-polarity molecules exhibited stronger antioxidant activity than higher-polarity fractions. Additionally, in terms of the antioxidant activity of the WP-derived peptide fractions, we observed that fractions containing low-polarity molecules exhibited more potent antioxidant activity than higher-polarity fractions. Therefore, the differential antioxidant activity of fractions owing to differences in polarity is a factor to consider in the application of WP hydrolysates.

Given that inflammatory response is one of the causes of various human diseases, proper inflammatory control is essential for the prevention and treatment of diseases. Specifically, treatment with WP had little effect on the LPS-induced expression of inflammatory cytokines in macrophages. However, treatment with all the WP hydrolysate fractions effectively suppressed the LPS-induced expression of inflammatory cytokines. Moreover, F6, the fraction with the lowest polarity, inhibited cellular senescence five times more effectively than WP. Our results confirmed the excellent anti-inflammatory and anti-cellular senescence effects of low-polarity fraction-contained peptides.

The radical scavenging activity of a peptide is affected by the hydrophobic amino acids constituting the peptide. Among the hydrophobic amino acids, methionine (M), proline (P), cysteine (C), alanine (A), glycine (G), valine (V) and leucine (L) are known to have a high radical scavenging ability. Further, lysine (K) at the C-terminus of the peptide improves the antioxidant ability (Zhu et al. 2013; Huang et al. 2017). The composition ratio of the hydrophobic amino acids of P1 and P2 detected in F6, the fraction with the lowest polarity, was approximately 40%, supporting the efficient radical scavenging activity of F6. The α-lactalbumin-derived P2 identified was a peptide which was confirmed by various proteolysis methods and is known to have an opioid effect on the cardiovascular system as well as excellent radical scavenging activity (Mullally et al. 1996; FitzGerald and Meisel 1999; Marcone et al. 2017). LC-MS analysis confirmed that P2 accounted for 16% of the various peptides included in F6 (Table 1). In order to confirm the contribution of the quantitative composition to various cellular activities of P2 in F6, when radical scavenging activity was compared representatively, at 5 mg/ml F6, about 55% (Figure 3), 0.84 mM (1 mg/ml) P2 at about 15% (Figure 6) activity was confirmed. These results indicate that P2 containing 16% in 5 mg/ml F6 was 0.8 mg/ml, and about 27% or more of the total radical scavenging activity of F2 was attributed to P2. The cell proliferation and radical scavenging activity of P2 can suppress cellular senescence and inflammatory responses caused by ROS, however, further studies on the molecular mechanisms underlying its activity through binding proteins and cell receptors are needed.

Overall, these results show that the hydrolysis of WP confers anti-inflammatory and anti-aging properties on a diversity of peptide molecules.

## Conclusion

In this study, we investigated the effects of hydrolysis, which increases the diversity of the bioactive peptides in WP, on several physiological activities of WP fractions. Reportedly, excessive oxidative stress causes various human diseases, including age-related diseases; (Kikuchi et al. 2002; Kennedy et al. 2005; Nunomura et al. 2006) however, the results of this study confirmed that through hydrolysis, WP fractions with strong antioxidant activity can inhibit the generation of ROS more effectively than WP. Moreover, we demonstrated that the WP fraction with the lowest polarity exhibited excellent efficacy with respect to cell proliferation, anti-inflammatory activity, and anti-aging activity. Therefore, the processing of WP fractions via hydrolysis has significance with respect to the discovery of novel bioactive functions and increasing the diversity of highly active bioactive peptides.
